# Carboxymethyl chitosan nanoparticle-modulated cationic hydrogels doped with copper ions for combating bacteria and facilitating wound healing

**DOI:** 10.3389/fbioe.2024.1429771

**Published:** 2024-09-20

**Authors:** Yaqin Li, Jianping Lu, Jingru Shi, Lingjiao Zhang, Haibo Mu, Tong Cui

**Affiliations:** ^1^ Xinjiang Xinhe Zhitong Technology Service Co. Ltd., Urumqi, China; ^2^ College of Chemistry & Pharmacy, Northwest A&F University, Yangling, Shaanxi, China; ^3^ Xinjiang Xinhe Zhitong Biotechnology Co. Ltd., Urumqi, China; ^4^ Karamay Central Hospital of Xinjiang, Karamay, China

**Keywords:** copper ions, carboxymethyl chitosan, hydrogel, wound infection, cell migration

## Abstract

The simultaneous administration of antibacterial treatment and acceleration of tissue regeneration are crucial for the effective healing of infected wounds. In this work, we developed a facile hydrogel (PCC hydrogel) through coordination and hydrogen interactions by polymerizing acrylamide monomers in the presence of carboxymethyl chitosan nanoparticles and copper ions. The prepared PCC hydrogel demonstrated effective bacterial capture from wound exudation and exhibited a potent bactericidal activity against methicillin-resistant *Staphylococcus aureus* (MRSA) and *Pseudomonas aeruginosa*. Furthermore, slow release of copper ions from the hydrogel facilitated wound healing by promoting cell migration, collagen deposition and angiogenesis. Additionally, the PCC hydrogel possessed excellent biocompatibility and hemostatic properties. The practical effectiveness of PCC hydrogel in addressing bacterial infections and facilitating wound healing was verified using a mouse model of MRSA-induced wound infections. Overall, this work presents a simple yet efficient multifunctional hydrogel platform that integrates antibacterial activity, promotion of wound healing, and hemostasis for managing bacteria-associated wounds.

## Introduction

The poor wound healing of wounds following severe traumatic injury, surgery, extensive burn, or chronic diseases remains an imposing clinical challenge (Blacklow et al., 2019; Chen et al., 2020). This process is typically hindered by wound infection caused by diverse bacterial strains such as *Staphylococcus aureus*, *Pseudomonas aeruginosa*, *Escherichia coli* and drug resistant bacteria, such as methicillin resistant *Staphylococcus aureus* (MRSA) (Huang et al., 2023; Louis et al., 2022; Wardlaw et al., 2012). The open, moist and exuding nature of the wound creates an optimal habitat for these bacteria that subsequently leads to severe tissue infection and delays wound healing (Huang et al., 2020). Furthermore, delayed differentiation of fibroblasts may also impede the process of wound tissue repair and regeneration, resulting in localized pain, swellings, and potentially life-threatening complications. Therefore, efficient wound management necessitates simultaneous antibacterial treatment alongside promotion of tissue restoration at the site (Qiu et al., 2020).

Copper (Cu) ions have been recognized as effective antibacterial agents with long-term inhibition of bacterial proliferation and low resistance development (Wang et al., 2016; Xu et al., 2020). The antibacterial efficiency of copper ions appears to increase proportionally with the concentration. Generally, the bactericidal effect of copper ions is time-dependent and requires a relatively protracted duration to eradicate bacteria due to the sluggish interaction between ions and bacteria (Milenkovic et al., 2017). Therefore, an additional carrier is necessary to enhance the interaction with bacteria for achieving high bactericidal effectiveness (Liu et al., 2020a; Sang et al., 2021). Furthermore, the presence of copper can stimulate the upregulation of vascular endothelial growth factor (VEGF) and extracellular skin protein, as well as promote wound healing through mechanisms including cell migration stimulation, collagen deposition facilitation, angiogenesis promotion, etc., (Diao et al., 2023; Kornblatt et al., 2016). However, an excessive amount of free copper ions may give rise to severe toxic side effects, such as debilitating neurodegenerative disorders, convulsive spasms, and even potential fatality. (Ning et al., 2015). To mitigate the toxicity concern, locally controlled release of copper ions represents a desirable strategy (Fowler et al., 2019; Xiao et al., 2017; Xiao et al., 2018).

Hydrogel has emerged as an exceeding contender for wound dressings as a result of its exceptional biocompatibility, hydrophilicity and intricate 3D porous structure resembling the extracellular matrix (Yang et al., 2021). Furthermore, hydrogel can form physical barriers at bleeding sites, thereby facilitating hemostasis, maintaining an optimal wound environment, and enabling oxygen permeation (Liang et al., 2019). Considering that cationic polymer easily interacts with both Gram-positive and Gram-negative bacteria via electrostatic forces, van der Waals forces, and hydrophobic interactions; polyacrylamide (PAM) readily interacts with the negatively charged bacterial surface owing to its abundant amino groups (Fu et al., 2022; Niu et al., 2021) However, PAM hydrogel alone is always crisp with inadequate mechanical properties for dressing applications. (Ferrag et al., 2021; Olaret et al., 2022). Carboxymethyl chitosan (CMCS), a chitosan derivative, demonstrates improved solubility, antibacterial properties, antioxidant abilities, biodegradability as well as heavy metal ion adsorption capabilities. It has found extensive usage in the biomedical realm such as drug delivery, tissue engineering, and so on (Pan et al., 2019; Zhang et al., 2021). The plentiful amino and carboxyl groups on CMCS can coordinate with specific inorganic ions leading to gelation of solutions followed by swelling or shrinkage changes of hydrogels (Cao et al., 2021; Jiang et al., 2022). We supposed that CMCS not only coordinates with Cu^2+^ but also contributes to the plasticity of PAM hydrogel.

**SCHEME 1:**
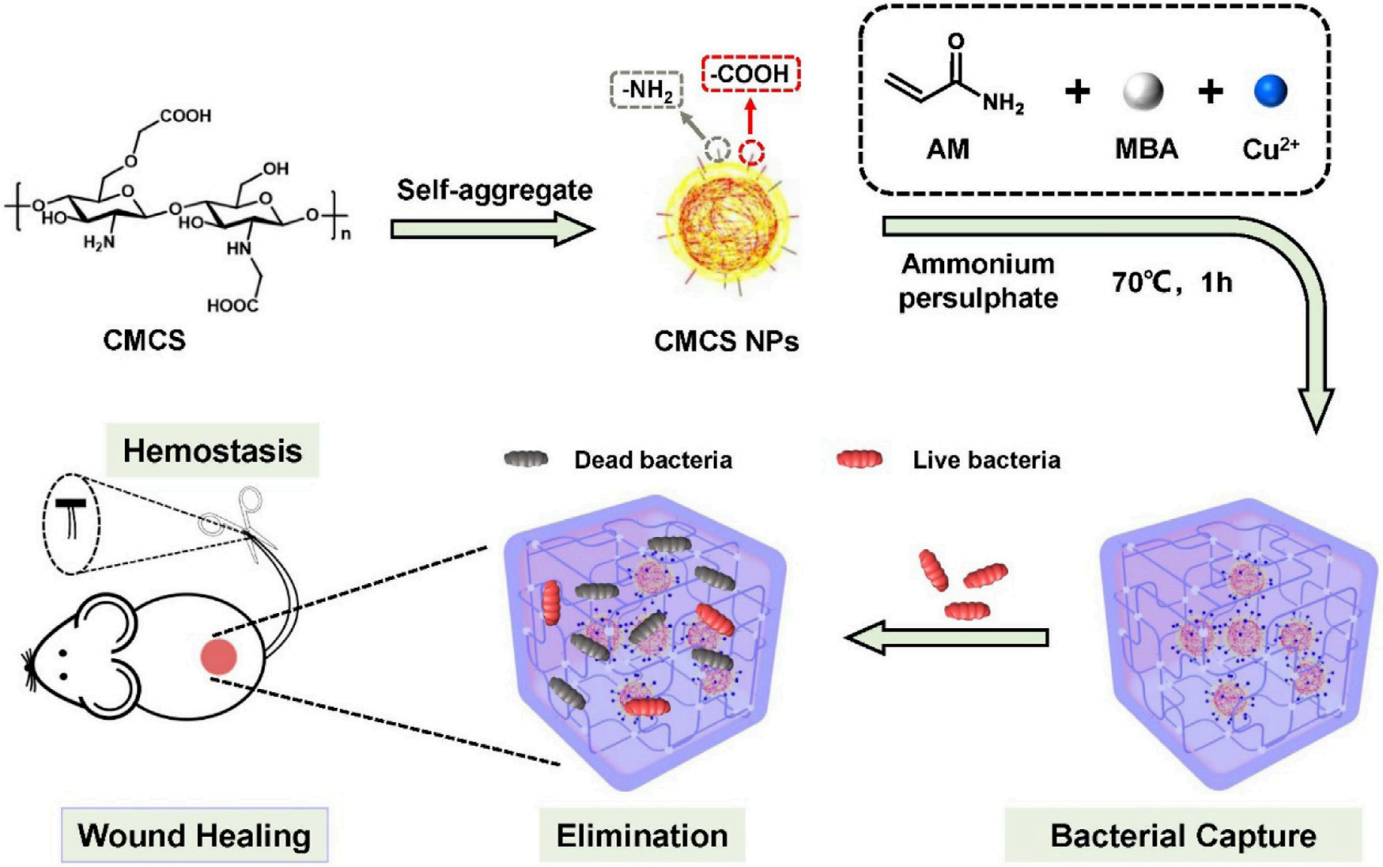
Schematics for the preparation of PCC hydrogel, and its envisioned potential in bacterial capture, eradication and facilitation of wound healing.

Inspired by the aforementioned facts, we designed a facile and biocompatible antibacterial hydrogel as a multifunctional wound dressing to combat bacterial infection and promote wound healing. As illustrated in Scheme 1, the PCC hydrogel was prepared by the polymerization of acrylamide monomers in the presence of Cu^2+^ and CMCS nanoparticles, facilitated by coordination and hydrogen interactions. In this PAM-Cu-CMCS hydrogel (PCC), Cu^2+^ readily formed coordination bonds with carboxyl and amino groups on both PAM chains and CMCS, resulting in slow release of copper ions to avoid excessive delivery and minimize potential toxicity risks. Simultaneously, the released copper ions exhibited enhanced wound healing properties by promoting cell migration, collagen deposition and angiogenesis at the wound sites. Moreover, the prepared PCC hydrogel effectively captured bacteria from wound exudation due to its interaction with bacteria via PAM moieties, thereby augmenting its bactericidal activity alongside copper ions. Additionally, this hydrogel demonstrated excellent hemostatic performance as an initial step towards efficient wound healing. Overall, our proposed PCC hydrogel formulation possesses remarkable biocapacities and antibacterial abilities that make it a promising candidate for managing infected wounds.

## Materials and methods

### Materials

Sodium carboxymethyl chitosan (CMCS, degree of substitution: ≥80%, product code C832672), acrylamide (AM), N, N′-methylenebis (acrylamide) (MBA), ammonium-persulphate (APS) were procured from Macklin (Shanghai, China). Calcium Chloride (CaCl_2_) and copper sulfate pentahydrate (CuSO_4_ 5H_2_O) were purchased from Kermel (Wuhan, China). Luria-Bertani (LB) broth and agar powder were obtained from AOBOX (Beijing, China). Bacterial strains including Methicillin resistant *Staphylococcus aureus* (MRSA) and *Pseudomonas aeruginosa* (PA) were purchased from BeNa Culture Collection (Beijing, China). L929 mouse fibroblast cell line was sourced from Gaining Biological (Shanghai, China). Dulbecco’s Modified Eagle Medium (DMEM), penicillin and streptomycin were obtained from Gibco (Beijing, China). Fetal bovine serum (FBS) and 3-(4,5-Dimethylthiazol-2-yl)-2,5-diphenyltetrazolium bromide (MTT) were acquired from Solarbio (Beijing, China). Propidium iodide (PI) was procured from Keygen biotech (Nanjing, China). Annexin V-FITC/PI kit and diamidino-2-phenylindole (DAPI) were purchased from BestBio (Shanghai, China). All other chemical reagents utilized for synthesis were of analytical grade and used without undergoing additional purification.

### Preparation of the PC and PCC composite hydrogel

The PCC hydrogel was synthesized via a free-radical polymerization and metal-ligand coordination approach (Jiang et al., 2022). Initially, CMCS (3.0 wt%) was dissolved in 2 mL of DI water to form self-aggregated CMCS nanoparticles (CMCS NPs) (Nguyen et al., 2017). Subsequently, a mixture contained AM (0.7 g), APS (0.01 g), MBA (0.02 g) and CuSO_4_.5H_2_O (0.08 g) in DI water (2.0 mL) was added to the above CMCS NPs solution with stirring followed by a sonication for 10 min. After being cultured at 70°C for 1 hour, a blue PCC hydrogel was obtained. The copper content was arranged according to the literature (Akalin and Pulat, 2020; Sarkar et al., 2015). The PC hydrogel was prepared as above without CuSO_4_, and the polyacrylamide (PAM) hydrogel without CuSO_4_ and CMCS NPs.

### Characterization of the hydrogels

The size distribution of CMCS NPs in the aqueous solution were investigated using dynamic light scattering (DLS, ZEN3600, MALVERN INSTRUMENTS LIMITED, Malvern, England). The morphology was examined using transmission electron microscopy (TEM, FEI Talos F200X, Hillsboro, United States). The freeze-dried hydrogel samples were used for scanning electron microscopy (SEM, Nano SEM-450, FEI, Hillsboro, United States). The chemical components were analyzed by EDS (EDAX, Mahwah, United States) with a voltage of 200 kV and a resolution of 136.65. Rheological measurement was performed using a rheometer (DHR-1, Waters, Milford, America). The mechanical properties of hydrogel were tested as the literature (Jiang et al., 2022).

### Swelling ratio of hydrogels

The swelling ratio of PCC was tested by a gravimetric method (Liu et al., 2020b). Briefly, the hydrogel samples of equal weights (Wi) were immersed in DI water at 37°C. At indicated intervals, the hydrogels were taken out and the excess surface water was eliminated with filter paper, then the weight of hydrogels (W_t_) were recorded. The swelling ratio was calculated using the following equation: Swelling ratio = (W_t_−W_i_)/W_i_ × 100%.

### Bacterial capture efficiency

The capture abilities of hydrogel to bacteria were evaluated against MRSA and PA as reported (Liu et al., 2020a). Briefly, bacteria suspension (5 mL, 10^8^ CFU mL^−1^ in PBS) was incubated with the hydrogel sample (0.1 g) at 37°C for 10 min, the optical density at 600 nm of the bacteria suspension was recorded by microplate reader (PERLONG, DNM-9612, Beijing, China) after removing the hydrogel sample. Bacteria suspension treated with equivalent PBS was used as a blank control. Additionally, the hydrogel samples were subjected to SEM imaging as following morphological characterization of bacteria.

### Antibacterial activity

The *in vitro* antibacterial activities of PCC hydrogel were evaluated against MRSA and PA using a spread plate method (Yu et al., 2019). The hydrogel sample pieces (10 mm in diameter, 3 mm in thickness) were irradiated by UV lamp for sterilization and then immersed in PBS for 1 hour to reach absorption equilibrium. Subsequently, the pieces were incubated in bacteria suspension (2 mL, 10^6^ CFU mL^-1^ in LB broth) at room temperature for 2 hours. The bacteria suspension incubated in PBS was blank control. Afterward, the hydrogel pieces were taken out while expelling the bacterial suspension. The obtained suspensions were diluted 100 times with PBS, and 40 μL of the diluted solution was spread on LB agar and incubated at 37°C for 24 h. The bacterial colony forming units (CFU) were recorded accordingly. The relative bacterial viability was calculated using the equation: Relative bacterial viability = Nt/Nc × 100%, where Nt represents the number of bacterial colonies formed in the hydrogel treatment group, and Nc represents that formed in the blank control group.

The antibacterial capacity was also assessed through an inhibition zone assay (Wang et al., 2022). Briefly, 200 μL of bacterial suspension (10^8^ CFU mL^-1^) was spread on LB agar followed by placing a hydrogel piece at its center and then incubated at 37°C for 24 h. The inhibition zones were calculated by measuring the diameter of each zone.

### Fluorescence staining

To assess the bacterial membrane integrity, a fluorescence staining assay was conducted as previously described (Zhang et al., 2019). In general, 100 μL of bacteria suspension (10^8^ CFU mL^-1^) in PBS was incubated with hydrogel for 2 h. Subsequently, the bacteria attached to the hydrogel were rinsed with PBS (900 μL). Afterwards, 10 μL of PI (10 μg mL^-1^) and 10 μL of DAPI (10 μg mL^-1^) was added to the bacterial solution (200 μL) and incubated in darkness for 15 min. Finally, all samples were visualized by a fluorescence microscope (Leica, DMi8, Wetzlar, Germany). DAPI stained all cells blue while PI stained dead cells red.

### Morphological characterization of bacteria

After treatment as antibacterial assay, the samples were washed with PBS and fixed with glutaraldehyde (2.5%) for 12 h, and subsequently dehydrated using graded ethanol. Following critical point drying, all samples were further coated with platinum by sputtering and imaged using SEM.

### Cytotoxic evaluation

The cytotoxicity of PCC hydrogel was evaluated following a reported method (Xiao et al., 2019). The L929 cells were cultured in DMEM medium supplemented with 10% FBS and 1% penicillin-streptomycin at a temperature of 37°C under a humidified atmosphere containing 5% CO_2_. The hydrogel pieces were immersed in PBS at 37°C for 24 h, followed by immersion of hydrogel (1 g) in DMEM medium (5 mL) at 37°C for another 24 h. Subsequently, the hydrogel was removed and the medium containing hydrogel extracts was collected (×1 dilution). Cell viability was determined using a MTT method (Jiang et al., 2022). L929 cells (5.0 × 10^3^ cells per well) were seeded in a 96-well plate and incubated overnight. The culture medium was then replaced with different dilutions of the hydrogel extract medium and the plate were incubated for 24 h. PBS served as a blank control (100% viability), while phenol (5 mg/mL) acted as a positive control. For live/dead staining, L929 cells were pretreated with hydrogel extracts (5-fold dilution) as above, and then stained using Annexin V-FITC/PI kit and visualization by a fluorescence microscope.

### *In Vitro* hemolysis assay

Hemolysis assay was conducted as a previously established method (Jiang et al., 2022). Initially, red blood cells (RBCs) were isolated from the fresh mouse blood, washed with saline for 5 times, and then resuspended in an equivalent volume of saline. Simultaneously, 1 g of the hydrogel was immersed in 5 mL of saline for 24 h to obtain hydrogel extracts after removing the gel matrix. Subsequently, 0.2 mL of RBC dispersion was gently mixed with 0.8 mL of hydrogel extracts. DI water was used as a positive control while saline as a negative control. All the samples were then incubated at 37°C for 4 h, and subjected to centrifugation (8,000 rpm for 5 min). The supernatant was collected and measured at a wavelength of 492 nm. The hemolysis ratio was calculated as the equation: Hemolysis ratio = (As−Anc/Apc−Anc) × 100%, where As represents absorbance of the sample, Anc represents that of the negative control, and Apc represents that of the positive control.

### Release profile of Cu^2+^ from hydrogel

PCC (0.1 g) was incubated in 5 mL of deionized water at 37°C. 0.5 mL of the solution was then taken out at an indicated interval and supplemented with the same volume of DI water. The amount of released Cu^2+^ was determined by Inductively Coupled Plasma-Atomic Emission Spectrometry (ICP-AES, Aglient 5110, California, United States) (Xiao et al., 2018).

### Cell migration experiment

Cell migration was investigated by incubating L929 cells with the hydrogel extracts as reported (Qiu et al., 2020). The cell seeding density was 2.0 × 10^5^ per well. Subsequently, a linear wound was created by scratching using a cell scraper. Following this, the cells were rinsed with DMEM medium and further incubated with hydrogel extracts for an additional 12 or 24 h. PBS served as the blank control. Cell visualization was performed using an inverted biological microscope (DMi8, Leica, Wetzlar, Germany). The migration area was calculated using following equation: Migration area = At−A0, where A0 represents the blank area at 0 h and At represents the experimental groups at t hour (t = 12 or 24 h).

### Wound healing efficacy

The female Kunming mice (5 weeks) were obtained from Tengxin Experimental Animal Co., Ltd. and housed in cages with sawdust bedding in holding rooms maintained at a temperature of 25 °C and a relative humidity of approximately 40% for 7 days. Subsequently, the mice were allocated into three groups in a random manner, with six mice per group, to receive different treatment: medical tape, PC hydrogel, and PCC hydrogel. The dorsal area of each mouse was subjected to the creation of a circular wound (10 mm in diameter), followed by immediate application of MRSA suspension (10 μL, 10^8^ CFU mL^-1^). The control group’s wound was dressed using medical a tape while the wounds in other groups were dressed with hydrogels pieces. To prevent interference from chewing behavior, the hydrogel dressing was covered and protected with an additional medical tape layer. The hydrogels and taps were changed daily after measuring the size of wound. After a 7-day treatment period, the mice were executed by cervical dislocation, and the wound tissues along with major organs (heart, liver, spleen, lung and kidney) were collected for hematoxylin-eosin staining. Masson staining and immunohistochemical staining were performed on the wound skin samples as well. The animal procedures were conducted in full compliance with all applicable ethical regulations and received approval from the Northwest A&F University Animal Care Committee (NWAFU-314020038).

### Hemostatic property

The hemostatic efficacy of PCC hydrogel was assessed utilizing a mouse-tail amputation model (Yu et al., 2019). The mouse tail was amputated 30% of the length and exposed in air for 15 s, then the wound was immediately bandaged with either gauze or hydrogel. The weight and duration of blood loss were meticulously documented for evaluation of the hemostatic performance.

The fresh whole blood from mouse containing citrate dextrose was utilized for the blood clotting test (Yu et al., 2019). Briefly, the recalcified blood (100 μL, 20 mM CaCl_2_) was applied onto the hydrogel samples and incubated at 37°C for 20 min followed by photographic record. Subsequently, the unattached blood cells were gently dispersed by addition of DI water (1 mL), ensuring minimal disruption to the clot. The absorbance (540 nm) of the dispersed blood cell suspension was measured and photographed. The equivalent blood incubated with water served as a blank control. The blood clotting index (BCI) was figured out using the equation: BCI (%) = (As/Ac) × 100%, where As was the absorbance value in the sample group and Ac was that in the control group.

Blood cell adhered to the hydrogels were visually inspected by applying whole blood onto hydrogel samples or gauze followed by incubation at 37°C for 5 min. After removing the floating blood cells by washing with saline, immobilization and dehydration processes were performed on hydrogel samples or gauze prior to SEM analysis (Jiang et al., 2022).

### Statistical analyses

The statistical analysis and graphing were performed with the Graphpad Prism 8.0 (GraphPad Software Inc., San Diego, United States). The quantitative results are reported as the mean values accompanied by their corresponding standard deviations, which have been derived from three repeated experiments. A two-tailed Student’s *t*-test was utilized to compare two experimental groups and a one-way analysis of variance (ANOVA) for multiple groups. *p*-value less than 0.05 was considered significant.

## Results and discussion

### Synthesis and characterization of PCC hydrogel

**FIGURE 1:**
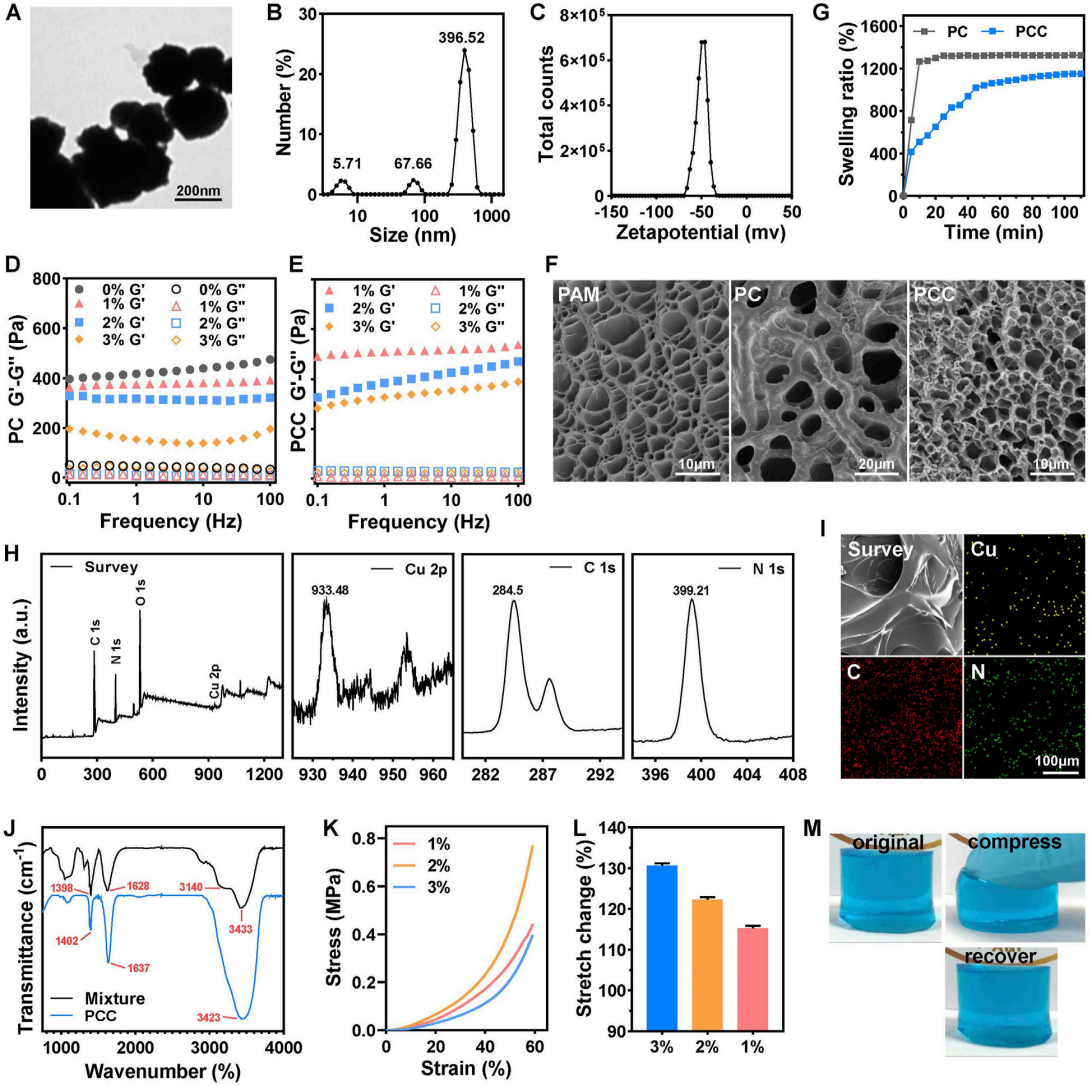
Characterization of PCC hydrogels. TEM image **(A)**, DLS **(B)** and Zeta potential **(C)** of CMCS NPs. The rheological properties of PC **(D)** and PCC **(E)** hydrogels with various content of CMCS NPs by performing an oscillatory frequency sweep at a strain of 0.1%. **(F)** The SEM images of PAM, PC and PCC hydrogels. **(G)** The swelling ratio of the PC and PCC hydrogels. (n = 3). **(H)** XPS spectra of PCC hydrogel included a comprehensive survey of all elements and high-revolution spectra of Cu 2p, C 1s, N 1s. **(I)** EDS elemental mapping of Cu, C and N in PCC hydrogel. **(J)** FTIR spectra of PCC hydrogel and mixture of components in PCC hydrogel. The compressive stress-strain curves **(K)** and stretch change **(L)** of PCC hydrogel with various content of CMCS NPs. **(M)** The original, compression and recovery of PCC hydrogel.

The CMCS NPs were synthesized using an ionic gelation method (Nguyen et al., 2017). The TEM image in Figure 1A showed the regular spherical morphology of CMCS NPs with a diameter ranging in several hundreds of nanometers. The DLS data revealed that CMCS NPs exhibited a hydrated size of 396 nm (Figure 1B) and a Zeta potential of −50 mV (Figure 1C). The hydrogel was synthesized by the free radical polymerization of acrylamide doped with Cu^2+^ and CMCS NPs via multiple physical crosslink (Scheme 1). In the hydrogel matrix, Cu^2+^ acted as coordination metal ions that easily complexed with carboxymethyl and amino groups of CMCS NPs and PAM molecular chains. The CMCS NPs and PAM chains were also able to crosslink by forming hydrogen bonding among the carboxymethyl groups and the amido or hydroxy groups (Pan et al., 2019). Moreover, the protonated amino groups on PAM chains or CMCS NPs interacted electrostatically with the deprotonated carboxymethyl groups on the CMCS NPs (Cao et al., 2021). The collective interactions mentioned above synergistically facilitate gelation and confer distinctive physicochemical properties of the prepared PAM-CMCS-Cu^2+^ hydrogel (denoted as PCC).

The tuned behavior of CMCS NPs towards hydrogel was initially investigated. The dynamic rheological measurements were conducted on PC hydrogel with varying concentrations of CMCS NPs, revealing a clear viscoelastic behavior characterized by the storage modulus (G′) being consistently higher than the loss modulus (G″) across the entire frequency range, indicating the formation of a robust hydrogel network (Figure 1D). PAM hydrogel without CMCS NPs (0%) showed the highest G′ value, suggesting superior mechanical properties under strain shock. In other words, PAM hydrogel was relatively rigid and brittle, which is unfavorable for wound dressing applications. However, incorporation of CMCS NPs effectively reduced G′ in a concentration-dependent manner within PC hydrogel. Similarly, higher content of CMCS NPs (3% w/v) ensured improved viscosity of PCC hydrogel (Figure 1E). For optimal dressing application suitability, 3% CMCS NPs was selected to prepare the subsequent studies’ hydrogels. In fact, PAM containing 3% CMCS NPs showed exhibited the lowest compression strength of 0.4 MPa at 60% of strain (Figure 1K) and the maximum tensile rate of 130% (Figure 1L). The cylindric PCC hydrogel could withstand certain-level deformation of compression and recover into the initial state instantly without any damage (Figure 1M). SEM images in Figure 1F showed distinct porous microstructures within PAM hydrogel resulting from acrylamide polymerization process while granulated CMCS NPs were uniformly embedded in PC hydrogel’s skeleton forming PAM-CMCS NP hybrid gel structure. Upon introduction of Cu^2+^, PCC exhibited a more compact three-dimensional porous microstructure, which has the potential to enhance air permeability within the gel matrix. The swelling ratio after 60 min for PCC exhibited a reduction of approximately 19% compared to that observed for PC alone (Figure 1G). This favorable swelling property and water absorption capacity make it an ideal candidate as an absorbent material for wound exudate management, aiming to minimize bacterial infections and facilitate efficient wound healing processes accordingly. The chemical composition of PCC hydrogel was analyzed using X-ray photon spectroscopy (XPS). As depicted in Figure 1H, PCC consisted of C, N, Cu and O elements. The peaks observed at 284.5, 399.21, and 933.48 eV correspond to the C1s, N1s, and Cu 2p orbitals, respectively. Elemental mapping in Figure 1I confirmed the homogeneous distribution of C, N and Cu within the PCC matrix. The FTIR spectra in Figure 1J revealed that the PPC hydrogel showed similar characteristic peaks from CMCS/AM/MBA mixture. For the mixture, the peaks at ∼3,433 and ∼3,140 cm^−1^ were attributed to the N-H asymmetric and symmetric stretching modes of −NH_2_ groups, respectively. The characteristic peaks at ∼1,628 cm^−1^ (C=O) could be assigned to the amide-I mode of the side amide groups of AM. The peak at ∼1,398 cm^−1^ was assigned to the symmetrical stretching of -COO- of CMCS. These above characteristic peaks shifted slightly for PCC hydrogel (Godiya et al., 2019). Taken together, the collective results demonstrated the successful synthesis of PCC hydrogel.

### Bacterial capture efficiency

**FIGURE 2:**
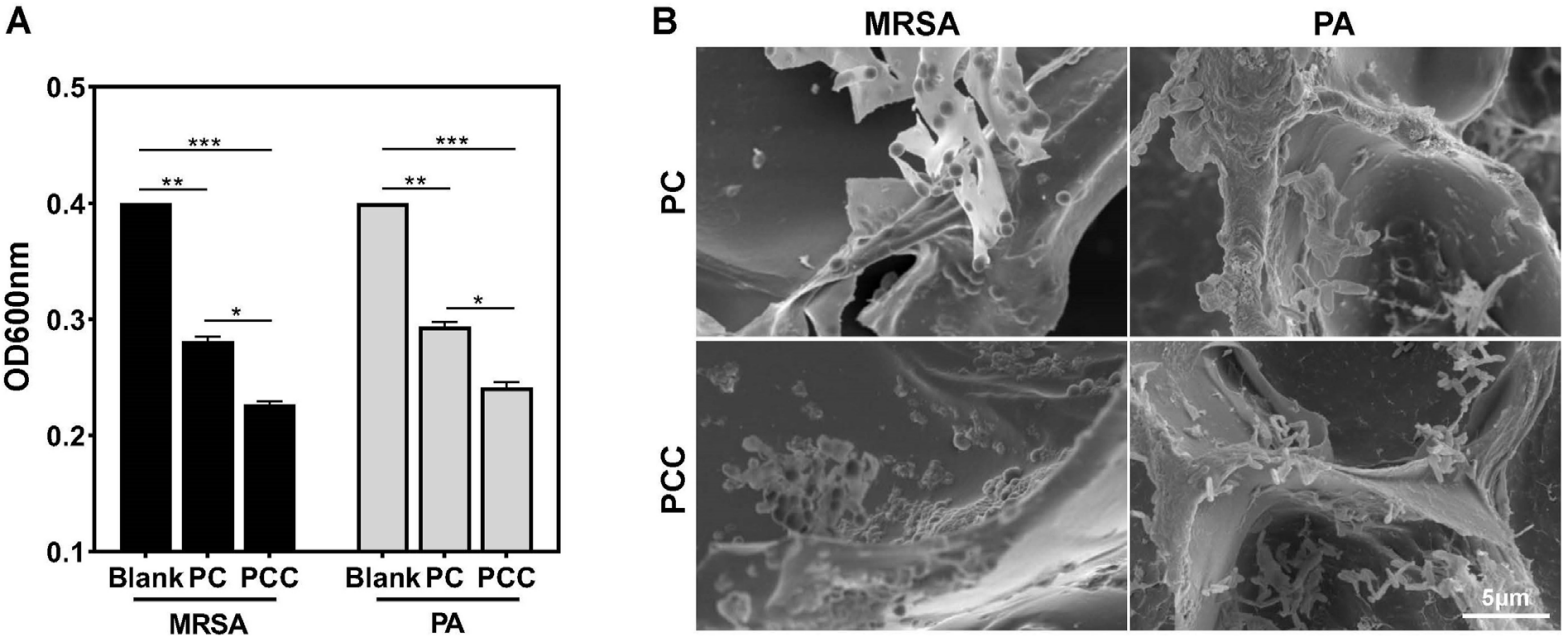
Bacterial capture efficiency. OD_600_ **(A)** and relative SEM images **(B)** of bacteria suspensions incubated with PC or PC hydrogels for 10 min. Data are presented as means and standard derivation (n = 3). Statistical significance is indicated by *p < 0.05, **p < 0.01, ***p < 0.001.

In view of the abundant amino groups present in acrylamides and CMCS NPs, which have been reported to interact with bacteria nonselectively through multiple interactions (Liu et al., 2020b), the bacterial capture efficiency was estimated *in vitro*. As shown in Figure 2A, after incubating the hydrogel with MRSA or PA suspension for 10 min, a significant decrease in OD600 value was observed. These observations cannot only be attributed to bactericidal ability because dead bacteria also possessed optical absorption (Jiang et al., 2022). Treatment with PCC induced a greater decline in OD600 compared to PC treatment, possibly due to the stronger interaction between Cu^2+^ ions and bacteria. SEM in Figure 2B further revealed that both PC and PCC hydrogel were able to effectively capture bacteria, and a higher number of bacteria were observed within the PCC hydrogel. These results confirmed that the prepared hydrogel can effectively capture bacteria, thereby enhancing bactericidal efficacy (Sang et al., 2021).

### *In Vitro* antibacterial activity

**FIGURE 3:**
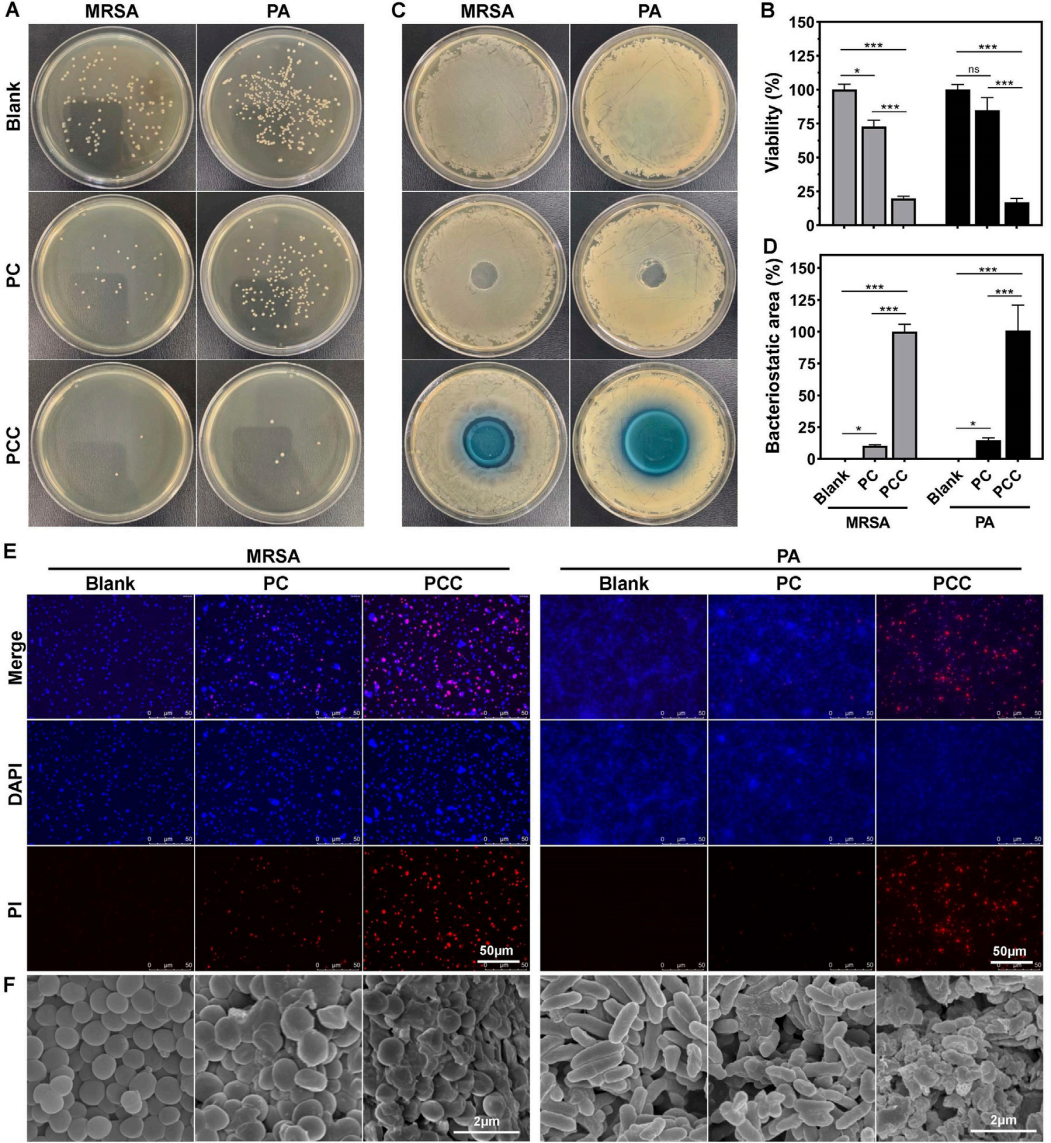
Antibacterial activity *in vitro*. Represent images of agar plates **(A)** and relative viability **(B)** of MRSA and PA following treatment with PC and PCC hydrogels. Photographs **(C)** and corresponding area percent **(D)** of inhibition zone. PCC treatment in MRSA or PA was normalized to 100% of inhibition zone. Fluorescence images **(E)** and SEM images **(F)** of bacteria treated with hydrogels. PI (red) indicated the dead bacteria and DAPI (blue) showed all bacterial cells. Data are presented as means and standard derivation (n = 3). Statistical significance is indicated by *p < 0.05, ***p < 0.001. ns, no significant difference.

MRSA is a typical strain of drug-resistant bacteria which have been widely used as a type strain in numerous of wound infection model (Xie et al., 2021). *Pseudomonas aeruginosa*, a common gram-negative bacterium known to adapt to harsh environments and antibiotics rapidly, cause chronic, antibiotic tolerant infections in wounds and lungs (Fleming et al., 2022). Therefore, we employed the two strains as models to investigated the *in vitro* antibacterial efficacy of PCC hydrogel. As shown in Figures 3A, B, PC hydrogel exhibited a moderate reduction in CFU counts, while PCC hydrogel effectively eradicated all bacteria with only a few remaining viable cells on the agar plate. Similar trends were observed in the inhibition zone test (Figures 3C, D), where PCC showed larger inhibition zones against both bacterial strains. These findings suggested that Cu^2+^ significantly enhances the bactericidal capacity of PCC hydrogel. Fluorescence imaging of bacteria was further utilized to estimate the antibacterial activity of PCC hydrogel. The dead bacteria were stained with red fluorescence by PI, while all bacteria were stained with blue fluorescence by DAPI. As illustrated in Figure 3E, the majority of bacteria exhibited a red stain following treatment with PCC, thereby indicating its inherent antibacterial capacity primarily attributed to the prolonged sterilization process and bactericidal ability conferred by Cu^2+^. Moreover, the surface morphology of bacterial cells was evaluated through SEM analysis. As shown in Figure 3F, bacterial cells from blank group displayed intact and smooth cell walls; however, upon treatment with PCC, significant alterations occurred as evidenced by wrinkled and fragmented cell walls. These results align well with the antibacterial mechanism involving Cu^2+^ ions and cationic antibacterial agents that induce disruption of bacterial membranes (Huang et al., 2020; Liu et al., 2020a).

### Biocompatibility and cell migration

**FIGURE 4:**
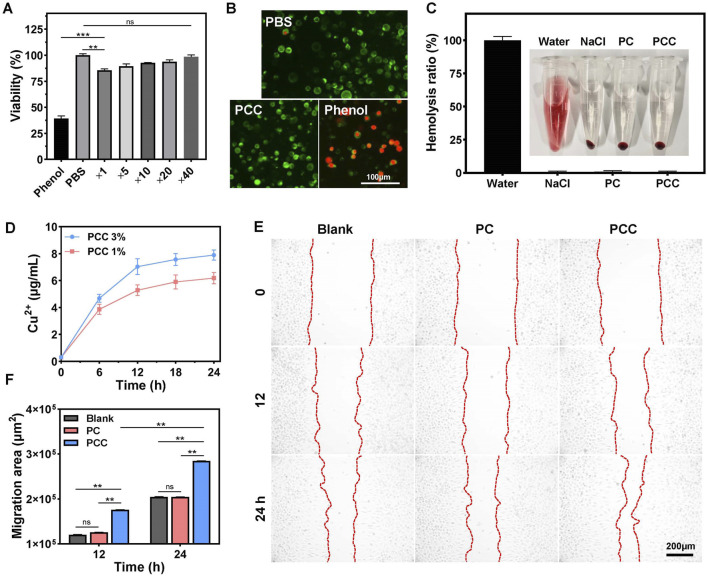
**(A)** Cell viability of L929 cells following different treatment: PBS, Phenol and PCC extract medium diluted for different times. **(B)** Live/dead staining images of L929 cells after treated with PBS, PCC hydrogel and phenol. **(C)** Hemocompatibility of hydrogels. Data are presented as means and standard derivation (n = 3). **(D)** The release profile of Cu^2+^ from PCC hydrogel. **(E)** The effects of PC and PCC hydrogels on L929 cell migration. **(F)** Migration area. Data are presented as means and standard derivation (n = 6). Statistical significance is indicated by **p < 0.01, *p* < 0.001. ns, no significant difference.

The cytotoxicity of the PCC hydrogel was evaluated using a standard MTT assay prior to its application in biomedical research. As indicated in Figure 4A, more than 85% of mouse fibroblast L929 cells remained viable following 24 h of incubation with PCC extract without dilution. To further evaluate cytocompatibility, a live/dead assay was conducted wherein the viable cells were stained with green fluorescence using calcein-AM and the dead cells were stained with red fluorescence using PI. As shown in Figure 4B, all the cells following treatment with PCC (5-fold dilution) exhibited green staining indicating their viability, similar to those with PBS treatment. The phenol (positive control) group, in contrast, demonstrated a near-total cell death and red labeling. Additionally, the hemocompatibility of PCC was evaluated by conducting a hemolysis assay. As presented in Figure 4C, the supernatants of saline, PC and PCC groups appeared transparent without any signs of red blood cell lysis. Conversely, the water group displayed bright red color due to complete hemolysis. These findings demonstrate that PCC exhibits excellent cytocompatibility which is crucial for its potential biomedical applications.

During wound healing process, migration of cells towards the center is essential for wound closure. It has been reported that copper ions can promote cell migration in small amounts (Diao et al., 2023; Xiao et al., 2017); therefore, we investigated whether PCC hydrogel influences cell migration behavior. As illustrated in Figure 4D, Cu^2+^ release from PCC occurred gradually over time-dependent manner. The concentration of Cu^2+^ after incubation for 12 h did not exceed 8 μg/mL that has been considered noncytotoxic to mammalian cells but still possesses desired antibacterial activity (Fowler et al., 2019; Ning et al., 2015; Qiu et al., 2020), which is also consistent to the above results in Figures 4A, B. The effect on cell migration by the PCC hydrogel was further explored. As depicted in Figures 4E, F, PC did not exhibit any significant promotion of cell migration in comparation to the blank control, while PCC demonstrated a time-dependent enhancement of cell migration towards the central region of the interspace, confirming the slow release of Cu^2+^ by PCC and its ability to facilitate cell migration. These findings collectively demonstrate that PCC possesses excellent biocompatibility and remarkable capacity for promoting cell migration, thereby highlighting its potential applications in wound healing.

### Hemostatic performance

**FIGURE 5:**
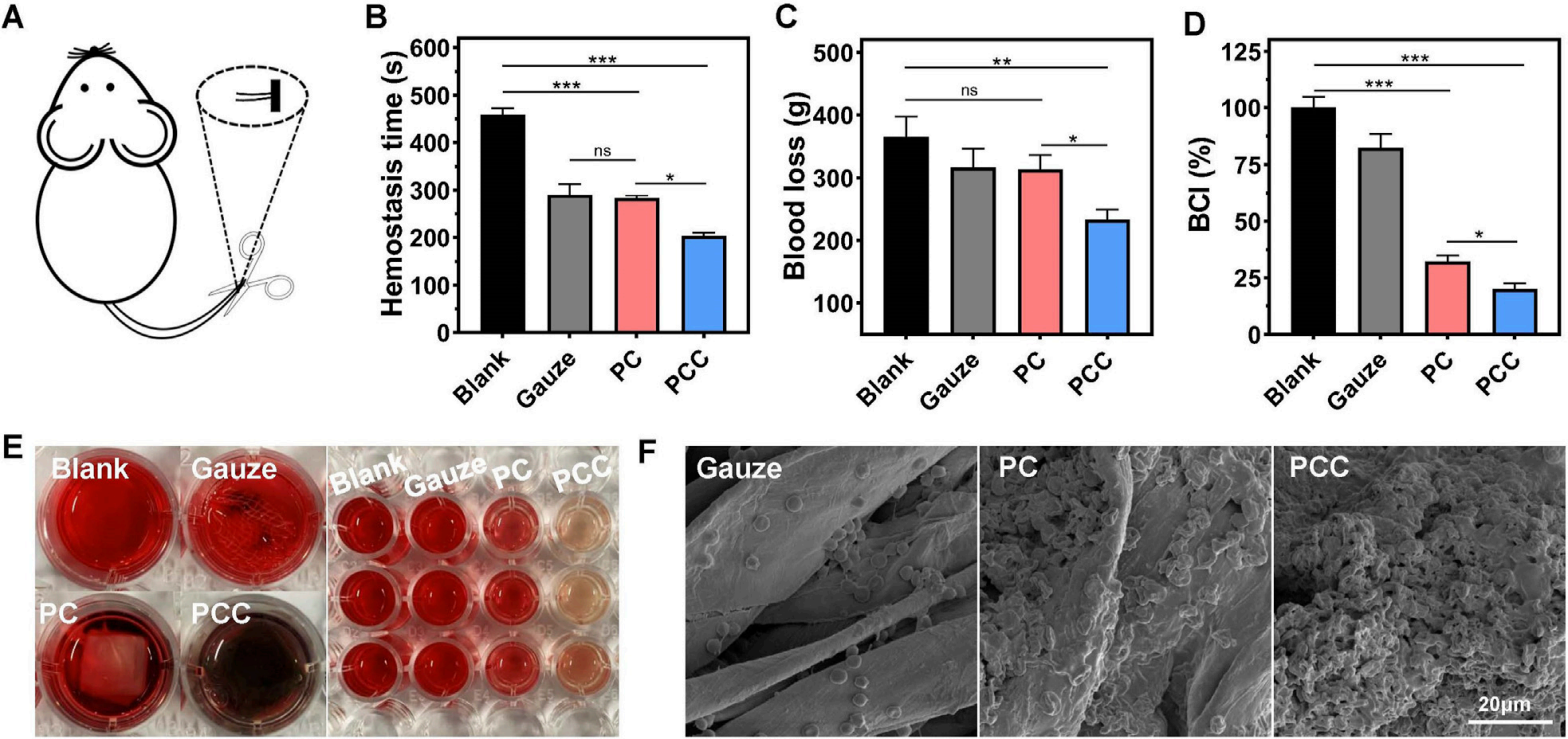
Hemostatic capacity of the hydrogels. **(A)** The hemostatic model was schematically depicted through the amputation of mice tails. The hemostatic time **(B)** and blood loss **(C)** of the model mice following treatment by gauze, PC and PCC hydrogels, and the group without any treatment was applied as a blank control. **(D)** BCI. **(E)** The represent images of different samples in whole blood clotting test. Left: blood cells incubated with water (blank), gauze, PC and PCC hydrogels. Right: the unattached blood cells dispersed by water following previous incubation. **(F)** The SEM images of hemocytes adhere to different samples. Data are presented as means and standard derivation (n = 3). Statistical significance is indicated by *p < 0.05, **p < 0.01, ***p < 0.001. ns indicated no significant difference.

Typically, wound healing progresses through four overlapping stages: hemostasis, sterilization, inflammation and remodeling. Therefore, for wound dressing materials, the evaluation of hemostasis is crucial due to the potential excessive blood loss resulting from trauma (Zhao et al., 2018). In this study, Kunming mice were used as an animal model by cutting down a portion of their tails (Figure 5A). Compared to the blank control or gauze groups, covering the cut with PCC significantly reduced both hemostatic time and blood loss (Figures 5B, C). There were not any significant differences in the hemostatic time and the amount of blood loss between PC and gauze treatment. To investigate the mechanism of hemostasis, the blood clotting index (BCI) was tested by dropping blood cells on different samples and incubation for 20 min followed by measurement of the OD540 value of unattached blood cells in water (Yu et al., 2019). Figures 5D, E showed that both PC and PCC exhibited a blood clotting index of approximately 25%, significantly lower than that of gauze group (80%). The lower index means fewer blood cells dispersed in water, signifying a higher efficacy in blood coagulation. In addition, the adhesion geometry of red blood cells on different samples were viewed using SEM images. As shown in Figure 5F, a substantial quantity of red blood cells adhered to PCC exhibiting abnormal or regular disk-like morphologies, while fewer cells were observed on PC surfaces. In contrast, the gauze surfaces exhibited minimal adherence of red blood cells. The collective findings suggested that PCC exhibits significant potential as a hemostatic material for wound dressing, owing to its remarkable ability to effectively cease bleeding.

### Evaluation of wound disinfection and healing

**FIGURE 6:**
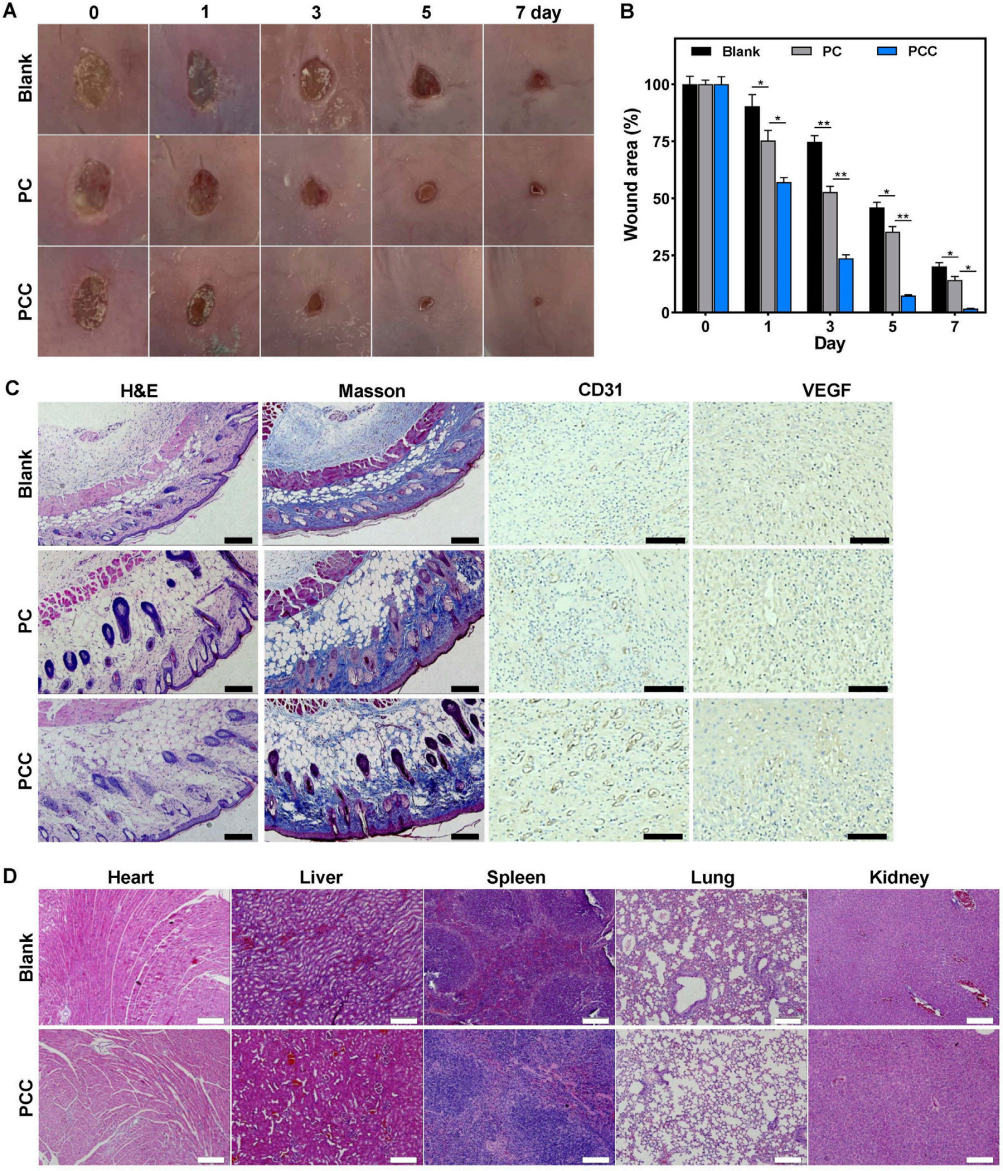
The therapeutic efficacy of hydrogel in MRSA-infected wound mice model. **(A)** Representative wound images. **(B)** The wound closure rates. **(C)** The H&E and Masson’s trichrome staining, and CD31/VEGF immunohistochemical staining of wound tissues following 7 days treatment. Scale bar: 100 µm. **(D)** The H&E staining of the major organs following 7 days treatment. Scale bar: 100 µm. Data are presented as means and standard derivation (n = 6). Statistical significance is indicated by *p < 0.05, **p < 0.01.

Encouraged by the remarkable characteristics of PCC hydrogel, we investigated its potential as a wound dressing using a mouse model with infected wounds. Round skin lesions with a diameter of approximately 10 mm was induced on the dorsal area of every mouse, followed by MRSA infection. The mice were then divided into three groups receiving distinct regimens: PBS, PC, and PCC hydrogel. The wounds were photographed at regular intervals of every 2 days (Figure 6A). The appearance of erythema and edema around the wounds was observed in all groups on day 0. However, compared to the control group, those treated with PCC exhibited significantly smaller wounds from day 1 onwards. After 7 days of treatment, all groups showed reduced wound areas compared to before treatment; notably, the mice that received PCC treatment exhibited minimal residual open wounds. The wound size further substantiated the superior efficacy of PCC over PC treatments in promoting wound healing (Figure 6B). Considering the process of infected wound healing (Qiu et al., 2020), it can be concluded that PCC’s excellent abilities to kill bacteria and promote cell migration are key factors contributing to the accelerated wound healing *in vivo*.

The wound healing process was further assessed through histological examination. The wound tissues form various treatment groups were subjected to H&E staining on day seven after the injury (Figure 6C). The control group demonstrated a notable presence of inflammatory infiltration and diminished dermis tissue. Conversely, the other two groups exhibited regenerated dermal tissues that contained newly formed skin appendages such as dermal fibroblasts, neovascularization, and hair follicles. The PC group still exhibited a considerable presence of inflammatory cells. The PCC group, in contrast, showed decreased inflammation and significant regeneration of dermis tissue, thereby indicating its superior therapeutic efficacy. The Masson’s trichrome staining was further utilized to demonstrate the deposition and organization of collagen fibers during the process of wound healing (Figure 6C). It was evident that the PCC group exhibited significantly higher levels of collagen content and denser deposition of collagen in the wound region. To detect the effect of PCC hydrogel on angiogenesis, platelet endothelial cell adhesion molecule-1 (CD31) and vascular endothelial growth factor (VEGF), two angiogenic markers, were detected in the wound tissue by immunohistochemical staining (Lin et al., 2020; Xiang et al., 2024). There were no significant differences regarding the expression of CD31 and VEGF in control and PC groups. However, more CD31 and VEGF positive cells were observed in PCC group (Figure 6C), which demonstrated that more intensive blood vessels were distributed in wound tissues in PCC group. These collective findings suggested that PCC demonstrated efficacy in promoting wound healing in MRSA-infected mice by effectively eliminating bacteria, promoting collagen deposition and angiogenesis. The H&E staining of organs in Figure 6D revealed no evident organ damage or inflammatory lesions, suggesting a negligible toxicity and high *in vivo* biosafety of PCC hydrogel.

## Conclusion

The present study successfully developed a PCC hydrogel that synergistically combines bacterial capture and acceleration of cell proliferation to treat bacteria induced wound infection. The PCC hydrogel was facilely prepared using a free radical polymerization of acrylamide doped with CMCS NPs and Cu^2+^ via multiple physical crosslinks. This hydrogel demonstrated effective bacterial capture and elimination capabilities, while also facilitating sustained release of copper ions to promote wound healing through enhanced cell migration, collagen deposition and angiogenesis. Furthermore, PCC demonstrated excellent biocompatibility and hemostatic properties. Ultimately, the hydrogel was effectively utilized as a wound dressing and exhibited remarkable efficacy in promoting wound healing in a mouse model. This work introduces a straightforward yet highly effective multifunctional platform against pathogenic bacteria, thereby demonstrating its immense potential in the field of wound management associated with bacterial infections.

## Data Availability

The original contributions presented in the study are included in the article/supplementary material, further inquiries can be directed to the corresponding authors.

## Abbreviations

CMCS NPs, carboxymethyl chitosan nanoparticles; PAM, polyacrylamide.
